# The effect of Chinese herbal medicine on male factor infertility: study protocol for a randomized controlled trial

**DOI:** 10.3389/fendo.2024.1418936

**Published:** 2024-07-22

**Authors:** Qidan Wen, Huanying Xu, Haoxi Zou, Pei Wang, Xiaoyan Xing, Ying Chen, Qiaoling Zhu, Yu Chen, Minhua Tan, Miaomiao Zhang, Ting Pan, Yanfen Chen, Yingju Wang, Suzhen Wu

**Affiliations:** ^1^ Foshan Clinical Medical School of Guangzhou University of Chinese Medicine, Foshan, Guangdong, China; ^2^ TCM Gynecology Department, Foshan Fosun Chancheng Hospital, Foshan, Guangdong, China

**Keywords:** Chinese herbal medicine, male factor infertility, live birth rate, semen quality, randomized controlled trial

## Abstract

**Background:**

The global prevalence of infertility is 9%, with male factors potentially accounting for 40% to 60% of cases. Conventional treatments can be ineffective, invasive, costly, and linked to adverse effects and high risks. Previous studies have shown that, Chinese herbal medicine (CHM) can regulate the hypothalamus-pituitary-testis axis, improve sperm abnormalities and quality, mitigate oxidative stress, and decrease DNA fragmentation index (DFI). Yet, the evidence backing the use of Chinese herbal medicine (CHM) for treating male factor infertility lacks conviction due to study design limitations, and there remains a scarcity of studies on the live birth rate following CHM treatment for male factor infertility. Here, we describe the rationale and design of a randomized waitlist-controlled trial to evaluate the effect of CHM on the live birth rate among males with infertility.

**Methods:**

This study is a single-center, randomized, waitlist-controlled study. A total of 250 couples diagnosed with male factor infertility will be enrolled in this study and then randomly allocated into two groups in a 1:1 ratio. Male participants in CHM group (treatment group) will receive CHM once a day for 3 months. Male participants in the waitlist group (control group) will not receive any treatment for 3 months. After 3 months, participants in both groups need to be followed up for another 12 months. The primary outcome will be the live birth rate; secondary outcomes include semen quality parameters, DFI and pregnancy related outcomes. Safety will also be assessed.

**Discussion:**

The purpose of this trial is to explore the effects and safety of CHM on the live birth rate among couples dealing with male factor infertility. The outcome of this trial may provide a viable treatment option for male factor infertility.

**Trial registration:**

Chinese Clinical Trial Registry: ChiCTR2200064416. Registered on 7 October 2022, https://www.chictr.org.cn.

## 1 Background

Infertility is defined as the failure to achieve pregnancy after 12 months of regular unprotected sexual intercourse (1). It has been reported that the worldwide fertility rates decreased from 4.7 to 2.4 live births between 1950 and 2017 (2). Additionally, there has been a parallel decrease in sperm counts (0.70 million/ml/year) noted between 1981 and 2013 (3). The global prevalence of infertility is 9% (4), with male factors potentially accounting for 40% to 60% of cases (5). Interference in any physiological stages of spermatogenesis or ejaculation can result in male infertility.

Although the conventional treatments such as medication, surgery, intrauterine insemination, and intracytoplasmic sperm injection have assisted numerous men with fertility issues in achieving clinical pregnancies (6–9). However, these treatments sometimes can be ineffective, invasive, costly or linked to adverse effects and high risks (10, 11). Therefore, it is essential to find a safe, convenient and effective method to treat male factor infertility.

Chinese herbal medicine (CHM), as an significant component of traditional Chinese medicine (TCM), has long been utilized to treat infertility in both women and men in China (12). The holism, together with syndrome differentiation and treatment, are the essence and the basic characteristics of TCM. Applying this theory to the treatment of male factor infertility has shown promising outcomes. According to TCM theory, kidney storing essence and plays a vital role in reproduction. Domestic studies have shown that kidney supplementing CHM can regulate the hypothalamus-pituitary-testis axis (13), regulate follicle-stimulating hormone (FSH) and luteinizing hormone levels bidirectionally (14–17), and finally improve the level of testosterone to improve semen quality. Furthermore, CHM can also prevent oxidative stress, reduce DNA fragmentation index (DFI) (18, 19), inhibit the apoptosis of germ cell (20–22), provide trace elements and vitamins (23, 24), and improve testicular microcirculation (25, 26).

However, there are some limitations in the existing clinical studies on CHM for male factor infertility, leading to insufficient evidence to be convincing. Most studies on CHM for male factor infertility are not randomized controlled trials, or do not provide detailed descriptions of random sequence generation and allocation concealment methods, and have small sample sizes. Furthermore, the lack of a unified evaluation standard for the efficacy of CHM has caused considerable heterogeneity. Therefore, well-designed randomized controlled trials are needed to acess the effectiveness of CHM in male factor infertility. Consequently, we have put forth a randomized controlled trial protocol to examine the influence of CHM on live birth rate among males with infertility.

The primary aim of this study is to access the hypothesis that CHM (treatment group) improve live birth rate more effectively than waitlist (control group) among males with infertility. Secondary objectives include the evaluation of semen quality parameters, sperm DNA fragmentation (SDF), pregnancy related outcomes, and adverse events.

## 2 Methods

This protocol was developed following the guidelines of the Consolidated Standards of Reporting Trials (CONSORT) (27) and adheres to the Standard Protocol Items: Recommendations for Interventional Trials (SPIRIT) statement 2013 (28) (**Additional File 1**). The trial obtained ethical approval from the ethics committee at Foshan Fosun Chancheng Hospital in China (ethics approval no. CYIRB-LCYJ-2022113). It has been registered at Chinese Clinical Trial Registry (ChiCTR2200064416).

### 2.1 Trial design

This is a single-center, randomized, waitlist-controlled clinical trial with 1:1 allocation ratio. The study will be conducted at Foshan Fosun Chancheng Hospital over a period of 24 months. All participants will be randomly allocated to receive CHM, once a day or waitlist for 3 months. After 3 months, participants in both groups will be followed up for 12 months. Investigators will screen based on the inclusion and exclusion criteria. This protocol aims to establish a well-designed randomized controlled trial to evaluate the effect of CHM for the improvement of live birth rate among males with infertility.

### 2.2 Participants and enrollment

**Figure 1:**
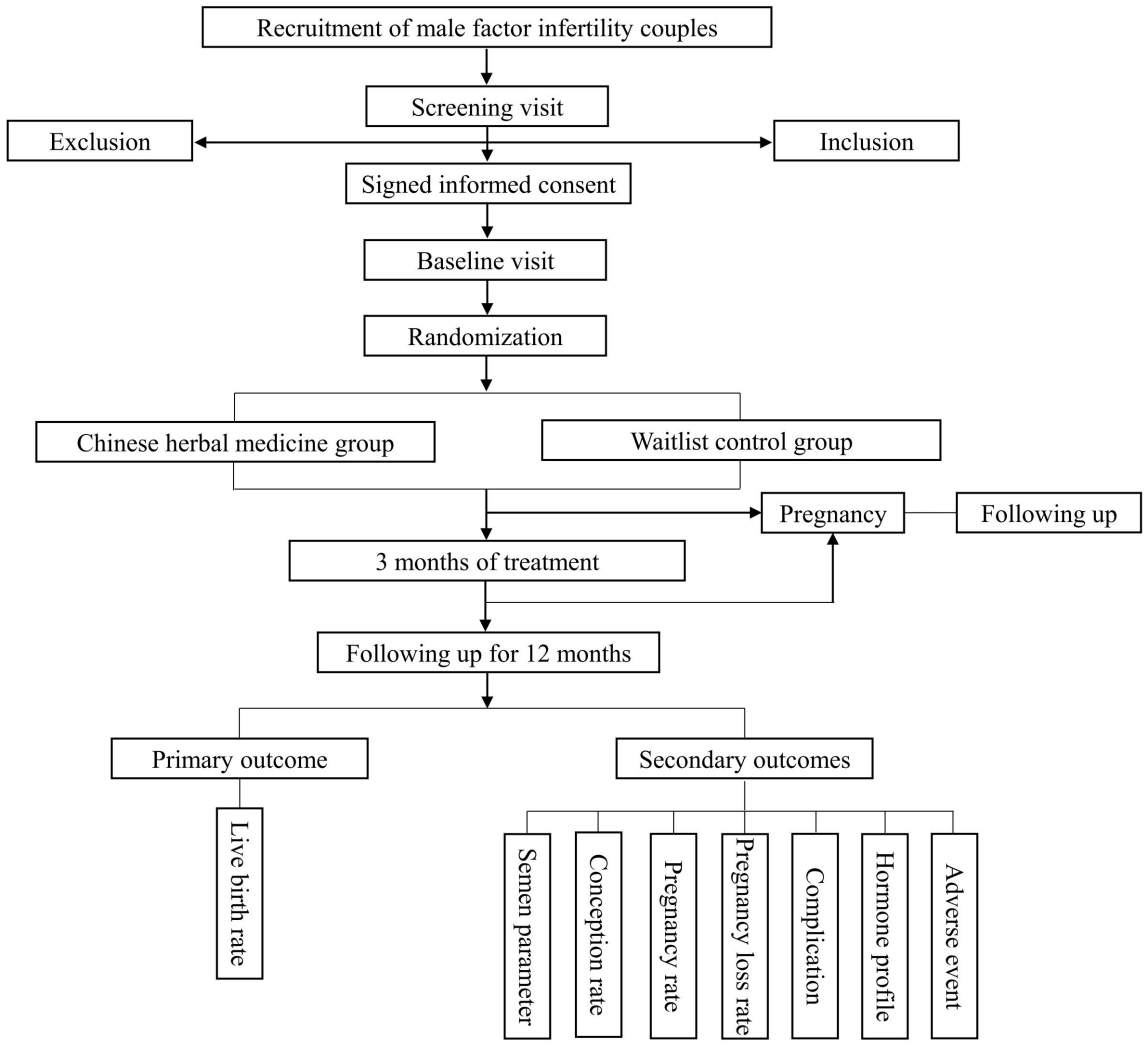
The flow diagram of the study.

Recruiting at least 12-month infertile couples who meet the inclusion criteria and do not meet any exclusion criteria from the Department of TCM Gynecology in the Foshan Fosun Chancheng Hospital. Eligible participants will be contacted and will be asked to sign the consent form following a thorough explanation of the study’s design and comprehensive counseling (see the flow diagram in **Figure 1**).

### 2.3 Inclusion criteria

(1) Couples inclusion criteria (29)

1) Infertility lasting 12 months or longer (whether primary or secondary);

2) Heterosexual;

3) Living together and able to have regular intercourse.

(2) Male inclusion criteria (29)

1) Older than or equal to 18 years old;

2) At least one of the following semen parameter deviates from the normal range in the semen analysis conducted over the past 6 months:

a. Sperm concentration ≤15 Million/ml;

b. Total motility ≤40%;

c. Normal morphology ≤4%;

d. DFI >25%.

3) Agree to provide semen samples as the proposed timeline at baseline, after 3 months of treatment, and after 3 months of follow-up;

4) Agree to sign the consent form.

(3) Female inclusion criteria (29)

1) ≥18 years and ≤40 years of age with normal ovarian reserve, defined as an early follicular phase FSH ≤10 IU/L, AMH ≥1.0 ng/ml, or antral follicle count greater than 10;

2) Regular menstrual cycle (25 to 35 days). Evidence to confirm ovulation, including biphasic basal body temperatures, positive ovulation predictor test, or serum progesterone level ≥3 ng/ml;

3) Evidence of at least one patent fallopian tube (determined by hysterosalpingogram or laparoscopy). 

### 2.4 Exclusion criteria

(1) Couples exclusion criteria (29, 30)

1) Previous history sterilization surgery, such as vasectomy or tubal ligation;

2) Planning to undergo *in vitro* fertilization within the next 15 months.

(2) Male exclusion criteria (29, 30)

1) Sperm concentration is less than 5 million/mL on screening semen analysis;

2) Present utilization of a medication or drug that would affect reproductive capabilities, for instance, anti-tumor and anti-epileptic medications;

3) Having taken CHM, Chinese patent medicine, or other treatments that could impact the research outcomes within the last three months;

4) Cryptorchidism or testicular dysplasia;

5) Genetic factors that have been discovered to lead to low fertility in males, including chromosomal disorders such as Y chromosome deletions;

6) Current poorly controlled chronic diseases diagnosed by a physician, such as heart disease, diabetes mellitus, hypertension, thyroid disorders, liver disease, kidney disease, or HIV/AIDS or cancer;

7) Unwilling to provide written consent for the study;

8) Patients who participated in other clinical studies requiring medication treatment.

(3) Female exclusion criteria (29, 30)

1) Diagnosed moderate or severe endometriosis;

2) Suffering from polycystic ovarian syndrome;

3) Body mass index greater than 35kg/m^2^;

4) Pregnant at present;

5) Currently suffering from serious medical illnesses such as cancer, heart disease, or cirrhosis

6) A history of receiving systemic chemotherapy or pelvic radiotherapy;

7) The medication or drug currently being used may affect reproductive function or metabolism.

### 2.5 Randomization and patient allocation

The statisticians at the Data Coordination Centre (DCC) will create the study’s randomization scheme using the Clinical Trial Management Public Platform (ResMan, www.medresman.org.cn). A total of 250 couples will be randomly assigned to either the treatment group or the control group in a 1:1 ratio. In this study, the treatment group will undergo CHM treatment, while the control group will not receive any medication, thus making it unachievable for researchers and participants to be blinded to the treatment allocation.

### 2.6 Intervention

Couples will receive complimentary ovulation predictor tests and guidance on the timing of intercourse throughout the study.

#### 2.6.1 Treatment group

The CHM used in this study contains 21 Chinese traditional medicinal herbs (**Supplementary Table 1**). The CHM prescription protocol is grounded in traditional Chinese medical theories. Following randomization, male partners in the treatment group will begin taking CHM once daily for a duration of 3 months. After a 3-month treatment period, participants will require a 12-month follow-up.

#### 2.6.2 Control group

During the study period, male partners in the control group will not receive drug intervention. After a 3-month waitlist period, participants will be followed up for 12 months.

### 2.7 Study-specific visits and procedures

Male participants will be required to attend several visits, which include the screening visit, baseline visit, treatment visit, and follow-up visit. Adverse events and concomitant medications will be documented at each visit. The overview of study visits is detailed in **Table 1**.

**Table 1:** Overview of study visits.

Period		Treatment				Follow-up	
Time point	Screening	basal	1m	2m	3m	1m ∼ 11m	12m
**Enrollment:**							
Eligibility screen	√						
Informed consent	√						
Allocation		√					
**Interventions:**							
Waitlist		√	√	√	√		
Chinese herbal medicine		√	√	√	√		
**Assessments:**							
Case history	√						
Vital signs	√	√	√	√	√	√	√
Anthropometric ^a^	√	√	√	√	√	√	√
Safety laboratory ^b^	√				√		√
Chromosomes of the peripheral blood	√						
Sex hormone steroids ^c^	√				√		√
Semen parameter analysis	√				√		√
Adverse event		√	√	√	√	√	√
Concomitant medications	√	√	√	√	√	√	√
Partner pregnancy test	√	√	√	√	√	√	√

^a^Anthropometric: weight, height, waist circumference, hip circumference. ^b^Safety laboratory: Blood routine test, thyroid function, renal and liver profile. ^c^Sex hormone steroids: Total testosterone, follicle stimulating hormone, luteinizing hormone, estradiol, progesterone, prolactin.

#### 2.7.1 Screening visit

(1) Obtain signed informed consent and detailed medical history collection;
(2) Semen analysis: semen analysis after 2–5 days of sexual abstinence included standard measurements, such as volume, pH, count, and motility. Semen samples will be assessed in the hospital laboratory using WHO 5.0 criteria (31) for sperm morphology. In addition, DFI will be evaluated using Sperm Chromatin Structure Analysis (SCSA) test (32);
(3) Laboratory examination: including reproductive hormone, blood routine test (BRT), thyroid function, liver function, kidney function, chromosome examination of peripheral blood.

#### 2.7.2 Baseline visit

After the screening visit, couples who meet the inclusion criteria will be assigned to the baseline visit and randomly allocated. Complete anthropometric (height, weight, hip circumference, waist circumference), vital signs measurement.

#### 2.7.3 Treatment visit

During the treatment period, visits were conducted at the end of each month, including the records of anthropometric, vital signs, compliance, adverse events and concomitant medication. At the end of the third month, the male partners in both groups will measure the following parameters within 1 week:

(1) Anthropometric: height, weight, hip circumference, waist circumference;
(2) Semen analysis, DFI;
(3) Laboratory examination: reproductive hormone, BRT, liver function, kidney function.

#### 2.7.4 Follow-up visit

Following the end of the final treatment, couples need to be followed up for another 12 months to observe the long-term effect of CHM on male factor infertility. The follow-up visit includes:

(1) Follow up whether the female partner is pregnant;
(2) At the end of the follow-up, the semen analysis and DFI of male partners will be rechecked.

#### 2.7.5 Pregnancy visit (only with conception)

Female partners who become pregnant within 15 months after randomization will have pregnancy visit, which will last until the termination of pregnancy (pregnancy loss or delivery). During the pregnancy visit, the following procedures will be completed.

(1) Blood human chorionic gonadotropin (HCG) test: pregnancy confirmation;
(2) Ultrasound examination (Conducted at 6–8 weeks of pregnancy): to access the quantity and of gestational sacs and the placement, as well as the dimensions, presence and size of fetal structures and to record the visualization of fetal heart motion and any pregnancy-related abnormalities;
(3) Record pregnancy loss: record biochemical pregnancy, ectopic pregnancy and all other intrauterine pregnancy loss (including spontaneous abortion, missed abortion, selective abortion and stillbirth);
(4) Record the confirmed pregnancy complications;
(5) After the female partner has given birth, the researcher will obtain a copy of the check list during pregnancy from the obstetrician to determine the pregnancy and delivery process of the female partner and the information of the newborn (whether it is a live birth, gender, weight, length, whether there is a congenital defect, etc.).

### 2.8 Adverse events

Participants involved in this study will not encounter major risks. The minor side effects of CHM may be dizziness, vomiting and diarrhea. The majority of these side effects are temporary and mild, which will disappear in a few days. During the study, the subjects will be asked about their drug tolerance, adverse events and serious adverse events will be recorded.

### 2.9 Outcome measures

#### 2.9.1 Primary outcome

Live birth rate: defined as a delivery taking place after gestation of ≥20 weeks.

#### 2.9.2 Secondary outcomes

(1) Semen parameters

1) sperm concentration: quantity of spermatozoa per unit of volume of semen, 10^6^/mL;

2) morphology: percentage of normal morphology;

3) motility: percentage of motile (including nonprogressive motile and progressive motile);

4) DFI: evaluate using SCSA test.

(2) Conception rate

Conception will be determined by a positive serum β-hCG measurement (β-hCG >10 mIU/mL).

(3) Pregnancy rate

Pregnancy will be characterized as an intrauterine pregnancy with confirmed fetal heart motion via transvaginal ultrasound.

(4) Pregnancy loss rate

Pregnancy loss will be refers to the intrauterine pregnancy lost before 20 completed weeks of gestation.

(5) Pregnancy complications

Including pregnancy diabetes, pregnancy induced hypertension, preeclampsia/preeclampsia, premature birth, congenital malformations, perinatal death, etc.

(6) Reproductive hormone

Including total testosterone, follicle stimulating hormone, luteinizing hormone, estradiol, progesterone, and prolactin.

(7) Adverse events.

### 2.10 Interim analysis

We propose abstain from conducting an interim analysis, and the final data analysis will be carried out after all live births in the trial.

### 2.11 Power calculation

Previous studies have shown that (33), among infertile men with abnormal semen, after taking placebo for 5 months, the live birth rate of spontaneous pregnancy of their female partners is 2.4%. It is assumed that in this study, the live birth rate of spontaneous pregnancy of couples in the waitlist-control group within 6 months after randomization is 2.5%, and 12.5% in the treatment group according to our clinical experience. Hence, the difference amounts to 10%, with two-sided α assigned to be 5% and β at 20% as the upper limit, while maintaining an 80% power and estimating a 15% dropout rate. Consequently, the sample size has been inflated from 105 to 125 per group, resulting in a total of 250 cases for the study.

### 2.12 Imputation procedure for missing data

We will document reasons for withdrawal in both randomization groups and qualitatively compare these reasons. The impact of any missing data on the results will be evaluated through sensitivity analysis using augmented data sets. To evaluate the effect of missing baseline data, we will employ the multiple imputation method with the missing-at-random assumption.

### 2.13 Data management

Upon enrollment in the study, each participant will be assigned a unique identifier, which will be utilized across all data records to maintain participant confidentiality. Participant data will be managed using paper case record forms (CRF) and a web-based electronic database (ResMan). Following the collection of original participant documents, they will be transferred to the CRF. Subsequently, all data will be stored in the ResMan. Quality control of the data will be managed at two distinct levels: the investigators will be responsible for ensuring data accuracy as the initial level of control during record input into the CRF. The second level will involve regular data monitoring and validation via ResMan by DCC. Any changes to the current study protocol will be presented to the institutional review board, all trial participants, and trial researchers. Ultimately, individual participant data, excluding private information, will be available to the public within 6 months after the trial’s completion.

### 2.14 Data monitoring and access

To ensure the safety of the study subjects, Data and Safety Monitoring Board (DSMB) will be established independently to review and interpret the data produced in this study. DSMB will oversee the study’s safety of and the integrity of data through routine video conferences or on-site inspections, offering recommendations on study conduct. In addition, DSMB will assess the advancement of the study, adjudicate adverse events, and determine on any potential premature closure of the study.

### 2.15 Auditing

Audit trail is another measure to ensure data quality and integrity. Authorization is required for any additions, deletions, or modifications of information in the electronic system record. This study will use computer-generated audit trail with time stamp to track changes in electronic source files. In addition, controls will be implemented to verify the accuracy of the date and time in the electronic system, with only authorized personnel permitted to make adjustments to the date or time. Once any abnormal system date or time is detected, they will be immediately notified. Internal security measures will be established in the computerized system. The data will be stored at the servers located at Foshan Fosun Chancheng Hospital, under the supervision and access of DCC. To prevent data loss, regular backup records will be conducted.

### 2.16 Data analysis

The outcomes were analyzed according to the intention-to-treat principle. The Kolmogorov–Smirnov test was used to test the normal distribution of continuous variables. Between-group comparisons were carried out by either a chi-squared test or Fisher’s exact test for categorical variables and by either Student’s t-test or Mann–Whitney U test for continuous variables. Comparisons of variables such as live birth rate, conception rate, pregnancy rate and pregnancy loss rate will include relative risk (RR) and 95% CIs in addition to the chi-squared test. A P-value <0.05 was considered to be statistically significant. All statistical analyses were performed using SPSS software version 23.0 (SPSS Inc., Chicago, IL, USA).

## 3 Discussion

The significant decrease in male fertility is closely related to factors such as unhealthy lifestyles and environmental pollution (34). The effectiveness of commonly used drugs for treating male infertility is still uncertain. Hormone therapy, antioxidant, antibiotics, corticosteroids, methylxanthine, vitamins, minerals and amino acids, and angiotensin converting enzyme inhibitors are the common drugs for treating male infertility (35). However, owing to inadequate controlled studies on potential therapeutic drugs or the absence of substantial enhancements in fertility, the United States Food and Drug Administration has not yet approved any drugs for treating male infertility. An analysis by Cochrane comprising 61 studies on male infertility indicated that antioxidant therapy might enhance the live birth rate, but the quality of evidence is inadequate and upon exclusion of studies at high risk of bias, the observed increase in the live birth rate was no longer apparent (36). A large multicenter randomized controlled trial showed that, compared with placebo, folic acid and zinc supplementation did not significantly improve sperm quality and live birth rate, and adverse reactions such as gastrointestinal tract increased (37). Similarly, another multicenter placebo-controlled randomized controlled trial also showed that antioxidants could not improve semen quality and partner pregnancy rate or live birth rate of male infertile patients (29). In addition, the application of hormone stimulation therapy in male infertility is still controversial due to its potential side effects (38). Therefore, it is crucial to identify a safe and effective treatment for male infertility.

Unlike the direct supplementation of certain hormone in Western medicine treatment, the CHM therapy emphasizes overall balance by enhancing the physical condition and regulating testicular function. TCM believes that the etiology of male infertility is multifactorial, and its pathogenesis is related to kidney, liver and spleen disorders. Therefore, the formulation principles of CHM in this study mainly include tonifying the kidney, soothing the liver and strengthening the spleen. Although studies have shown that traditional CHM can improve male semen quality, few studies include data on pregnancy outcomes, especially the lack of live birth rate, which is a key outcome of male factor infertility. Thus, the findings of this study could offer supportive evidence for the use of CHM in future clinical practice in this field.

## Trial status

The recruitment of participants has started in November 2022 and is going. The first version of the protocol was finished on June 30, 2022. After numerous discussions and amendments by the authors, there are 3 versions of the protocol. The enrollment of the current study will be conducted till December 2024.

## Data availability statement

The datasets used and/or analyzed after completing the current study will be available from the corresponding author by reasonable request.

## Ethics statement

The trial received ethics committee approval from Foshan Fosun Chancheng Hospital, China (ethics approval no. CYIRB-LCYJ-2022113). It has been registered at Chinese Clinical Trial Registry (ChiCTR2200064416). Written informed consent will be obtained from all participants. All procedures in the study are in accordance with the Declaration of Helsinki. The findings of this trial will be disseminated in peer-reviewed journals and presented at conference.

## Author contributions

QW: Writing – original draft, Writing – review & editing. HX: Writing – original draft, Writing – review & editing. HZ: Data curation, Investigation, Methodology, Writing – original draft. PW: Methodology, Writing – original draft. XX: Methodology, Writing – original draft. YiC: Data curation, Writing – original draft. QZ: Data curation, Writing – original draft. YuC: Data curation, Writing – original draft. MT: Data curation, Writing – original draft. MZ: Formal analysis, Writing – original draft. TP: Validation, Writing – original draft. YaC: Validation, Writing – original draft. YW: Validation, Writing – original draft. SW: Funding acquisition, Supervision, Writing – original draft, Writing – review & editing.
