# Supplementary material for: The effect of Chinese herbal medicine on male factor infertility: study protocol for a randomized controlled trial
Source: Front Endocrinol (Lausanne). 2024 Jul 22;15:1418936. doi: 10.3389/fendo.2024.1418936 (PMC11298452; doi:10.3389/fendo.2024.1418936)
Supplement: Supplementary file 1 [file Table_1.docx]

**Supplementary Table 1. Name and daily dose of Chinese herbal medicine used in the study.**

| **Name** | **Daily dose/g** |
| --- | --- |
| Astragali radix | 50 |
| Herba Cynomorii | 15 |
| Rhizoma Curculiginis | 15 |
| Radix Scrophulariae | 15 |
| Radix Codonopsis | 15 |
| Cortex Phellodendri | 10 |
| Pericarpium Citri reticulatae | 10 |
| Rhizoma Atractylodis | 10 |
| Radix Ophiopogonis | 10 |
| Radix Angelicae Sinensis | 10 |
| Radix Bupleuri | 10 |
| Fructus Amomi | 5 |
| Radix Glycyrrhizae | 10 |
| Rhizoma Cimicifugae | 10 |
| Rhizoma Atracylodis | 10 |
| Endothelium Corneum Gigeriae Galli | 10 |
| Semen Sojae Preparatum | 15 |
| Poria With Hostwood | 15 |
| Medicated Leaven | 15 |
| Radix Rehmanniae Recen | 15 |
| Actinolite | 15 |
